# Effectiveness of low-level laser therapy for patients with carpal tunnel syndrome: design of a randomized single-blinded controlled trial

**DOI:** 10.1186/1471-2474-13-248

**Published:** 2012-12-13

**Authors:** Rafael Inácio Barbosa, Eula Katucha da Silva Rodrigues, Guilherme Tamanini, Alexandre Márcio Marcolino, Valéria Meirelles Carril Elui, Rinaldo Roberto de Jesus Guirro, Nilton Mazzer, Marisa de Cássia Registro Fonseca

**Affiliations:** 1 Rehabilitation and Functional Performance Program, Faculty of Medicine of Ribeirão Preto, University of São Paulo, Avenida Bandeirantes, 3900, Ribeirão Preto, SP CEP 14049-900, Brasil; 2 Department of Neurology, Psychiatry and Medical Psychology, Faculty of Medicine of Ribeirão Preto, University of São Paulo, Avenida Bandeirantes, 3900, Ribeirão Preto, SP CEP 14049-900, Brazil; 3 Department of Biomechanics, Medicine and Rehabilitation of the Locomotor Apparatus, Faculty of Medicine of Ribeirão Preto, University of São Paulo, Avenida Bandeirantes, 3900, Ribeirão Preto, SP CEP 14049-900, Brazil

**Keywords:** Carpal tunnel syndrome, Low-level laser therapy, Rehabilitation

## Abstract

**Background:**

Carpal tunnel syndrome is the most common neuropathy in the upper extremity, resulting from the compression of the median nerve at wrist level. Clinical studies are essentials to present evidence on therapeutic resources use at early restoration on peripheral nerve functionality. Low-level laser therapy has been widely investigated in researches related to nerve regeneration. Therefore, it is suggested that the effect of low-level laser therapy associated with other conservative rehabilitation techniques may positively affect symptoms and overall hand function in compressive neuropathies such as carpal tunnel syndrome. The aim of this study is to evaluate the effectiveness of low-level laser therapy in addition to orthoses therapy and home orientations in patients with carpal tunnel syndrome.

**Methods/Design:**

Patients older than 18 years old will be included, with clinical diagnosis of carpal tunnel syndrome, excluding comorbidies. A physiotherapist will conduct intervention, with a blinding evaluator. Randomization will be applied to allocate the patients in each group: with association or not to low-level laser therapy. All of them will be submitted to orthoses therapy and home orientations. Outcome will be assessed through: pain visual analogic scale, Semmes Weinstein monofilaments™ threshold sensibility test, Pinch Gauge™, Boston Carpal Tunnel Questionnaire and two point discrimination test.

**Discussion:**

This paper describes the design of a randomized controlled trial, which aim to assess the effectiveness of conservative treatment added to low-level laser therapy for patients with carpal tunnel syndrome.

**Trial registration:**

Brazilian Clinical Trials Registry (ReBec) - 75ddtf / Universal Trial Number: U1111-1121-5184

## Background

Carpal tunnel syndrome (CTS) is the most common neuropathy in the upper extremity, resulting from the compression of the median nerve at wrist level. The compression is related with increased pressure at the carpal tunnel, elicited by the expansion of the structures contained within. Inflammatory or hemorrhagic conditions affecting the wrist, polyneuropathies, diabetes, rheumatoid arthritis, hypothyroidism, pregnancy and other hormonal alterations are also related with CTS [1-3].

The diagnosis of CTS is based on patient history and specific physical examination, and confirmed by complementary exams such as electroneuromyography. It affects mostly women in their fourth to seventh decade, presenting painful numbness at the median nerve path, often relieved with shaking the hands repeatedly (Flick’s sign) [4,5].

In order to properly assess the relief of symptoms and functional outcomes after conservative or surgical treatment of CTS, health professionals search for evaluation methods that are valid and reliable. Objective and subjective parameters must be taken into account, like measurements of pain, grip and pinch strength, sensory assessment, dexterity and patient perceived outcomes, through self-reported measures like the Boston Carpal Tunnel Questionnaire (BCTQ) [6]. The association of objective and subjective measures provides a better understanding about the physical and functional impact of CTS [7,8].

There are numerous instruments for sensory evaluation of the hand described in the literature [9-13]. Of all methods available, two point discrimination (2PD) and assessment of touch thresholds with Semmes Weinstein Monofilaments are most widely used in patients with CTS [9].

Pinch strength can be accessed through a dynamometer, such as the Pinch Gauge^TM^. Patients with CTS usually present diminished pinch strength, mostly in severe cases. The assessment of pinch strength can also help with determination of CTS severity degrees [14]. Amongst the available pinches to be assessed, pulp-to-pulp pinch seems to be the most responsive for CTS patients, due to the fact that is performed by muscles innervated by the median nerve [15].

The conservative treatment of CTS is based on non-steroidal anti-inflammatory drugs, local steroid injections and wrist splinting [16]. Other modalities like exercise therapy, therapeutic ultrasound and low level laser therapy are also described in the literature, being commonly used for the conservative treatment of CTS [17].

Patient education, with orientations about body posture while performing different activities of daily living are an important part of the rehabilitation program for conservative treatment of CTS. The patient must avoid repetitive wrist movements; especially wrist flexion associated with prolonged grip activities. Patient education booklets have showed great efficacy as a complement of treatment in many upper limb disorders [18].

Different types of wrist orthoses with variations in immobilization angles are reported in the literature as effective for CTS treatment. Orthoses is the most widely used method of conservative treatment, and its effectiveness is based on the principle that adequate positioning the wrist minimizes the compression of the median nerve, maintaining an optimal position to preserving the volume of the carpal tunnel and providing rest to the structures that go through the wrist, thus relieving the symptoms of CTS. The orthoses should be worn at night, although for patients with severe symptoms it can be also used during the day (full time or intermittent). In certain cases a orthoses that also immobilizes the metacarpophalangeal joints with 20 to 40 degrees of flexion can be prescribed, in order to minimize painful symptoms due to lumbrical muscles hypertrophy that contributes to the increased pressure at the carpal tunnel [19].

Among the available resources, studies with low level laser therapy (LLLT) demonstrated effectiveness for conservative treatment of CTS, probably due to LLLT biophysical effect in neural tissue that can facilitate its regeneration [20].

Some studies have focused on LLLT application in peripheral nerve injuries in the last decades. The first report of LLLT use with this purpose dates from the late 70’s by Rochkind et al., and since the early 80’s the scientific interest in this subject has increased, with papers demonstrating positive effects of LLLT in nerve cells regeneration [21,22]. From this moment on, other experimental studies were conducted in order to understand the role of that therapeutic modality in peripheral nerve regrowth and functional rehabilitation after nerve injuries [23-28]. Therefore, it is suggested that the effect of LLLT associated with other conservative rehabilitation techniques may positively affect symptoms and overall hand function in compressive neuropathies such as CTS.

## Methods/Design

The trial is a prospective randomized clinical trial, adhered to CONSORT guidelines, with the objective of assessing the effectiveness of conservative treatment with LLLT for patients with CTS, associated or not with other modalities like wrist orthoses and a patient education booklet.

### Ethics

The present study was approved by the Ethics Committee at the Hospital Clinics Faculty of Medicine of Ribeirão Preto (process number 7107/2010). All subjects recruited will sign an informed consent form.

### Eligibility criteria

**Figure 1:**
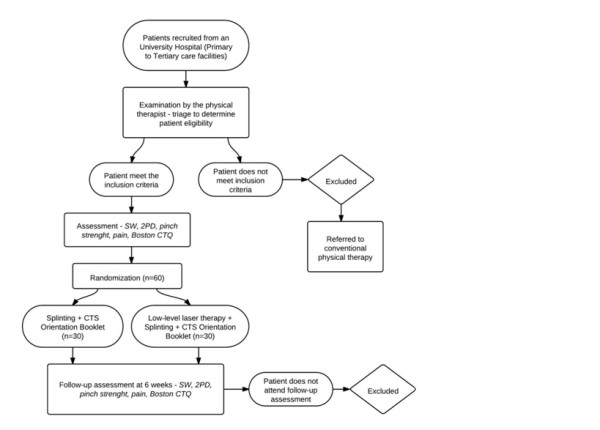
Carpal tunnel syndrome treatment flowchart.

Eligibility criteria are age over 18 years, clinical diagnosis of CTS, with no history or current associated upper limb injuries due to orthopedic, neurologic or rheumatologic disorders (Figure 1).

### Recruitment

Sixty patients with clinical and Electroneuromyography (EMG) diagnosis of CTS referred from a University Hospital will be recruited for our study. After an initial assessment, all patients that fit the inclusion criteria will be randomized to two different groups through a computer-generated list unknown to the recruiters, as follows:

• Control group. Conservative treatment with night splinting and patient education for 6 weeks.

• Low-level laser intervention group. Conservative treatment with night splinting, patient education and low-level laser therapy for 6 weeks (12 therapy sessions).

Hand surgeons will conduct clinical diagnosis and an experienced hand therapist will assess the outcomes, whose will be blinded to group assignment.

### Outcome measures

Assessment will be conducted pre and post intervention based on items related to pain, sensibility, functionality, symptoms and pinch muscle strength. Pain will be assessed by the pain visual analogic scale, sensibility by Semmes Weinstein monofilaments (Sorri^TM^, Bauru, Brazil) to detect threshold cutaneous sensibility and gnosis tactile through 2PD (North Coast, USA). Isometric pinch strength will be assessed through a dynamometer, Pinch Gauge ^TM^ (North Coast, USA). BCTQ [6] will be used as a specify disease functional and symptom related self-reported measure.

### Interventions

An experienced hand therapist will perform all interventions, in Rehabilitation Center of Clinical Hospital of the Medical School of Ribeirão Preto, University of São Paulo, Brazil.

#### Home orientations

##### Patient education booklet

A patient education booklet will be written specially for this study. It contains home and work orientation with emphasis in preventing activities which put the wrist in risk of median nerve compression at carpal tunnel, like excessive flexion and repetitive movement with the fingers or prolonged isometric sustained grip.

#### Wrist orthoses

**Figure 2:**
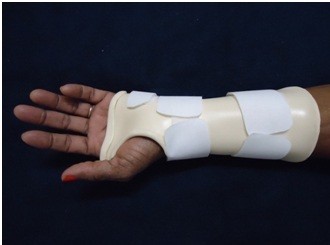
Wrist neutral immobilization splint, type 0.

The prescribed orthoses design will be in neutral wrist position, called immobilization splint, type 0, with the aim to reduce carpal intratunnel pressure and prevent from flexion posture (Figure 2), during night for 6 weeks.

#### Low-level laser therapy

A gallium-indium-phosphorus-aluminum (AlGaInP) laser emitter – Laserpulse^TM^, will be used (Ibramed™, Amparo, Brazil), with the following parameters: with wavelength 660 nm, mean power 30 mW, continuous regime and area of bean of 0, 06 cm^2^. The laser irradiation will be done with fluency of 10 J/cm^2^, energy (E) of 0.6 J, and exposure time of 20s for point, totaling six points of irradiation over the carpal tunnel. The laser will be positioned at an angle of 90° to the skin, according to the contact point technique. The patients will be irradiating twice a week, for 6 weeks (12 therapy sessions).

Gauging the laser emission will be conducted initially and after completion of the experiments.

### Statistical analysis

#### Data-analysis

A linear mixed-effects model will be used to compare the results. This model assumes individuals as random effects and groups as fixed effects, using time variation and interaction between them for calculating mean, standard deviation, and coefficient of variation. A 95% confidence interval and 5% significance level will be adopted. Linear mixed-effect models (random and fixed effects) are typically used for the analysis of grouped data from the same individual when independence supposition between observations within the group is not adequate.

This model assumes that the residual value from the difference between the predicted and observed values has a normal distribution, with zero mean and constant variance. In those situations where this supposition was not observed, the variable response was converted. The model was adjusted with SAS version 9.0 software.

## Discussion

Despite the widespread use of LLLT as one of the most popular and commonly used modalities in the field of physiotherapy, there is still limited evidence of its effectiveness. Only a few RCTs have investigated the effect of LLLT in treating patients with CTS; often with varying methodology qualities and have not been able to provide evidence regarding its usefulness.

The advantages of this study would be comparing the LLLT for patients with CTS, associated or not with other modalities like wrist orthoses and a patient education booklet to provide some evidence regarding this indication and use.

## Abbreviations

CTS: Carpal Tunnel Syndrome; BCTQ: Boston Carpal Tunnel Questionnaire; 2PD: Two point discrimination; LLLT: Low level laser therapy; EMG: Electroneuromyography; AsGaIP: Gallium– indium-phosphorus-arsenide; RCT: Randomised clinical trials.

## Competing interests

The authors declare that they have no competing interests.

## Authors’ contribution

The primary authors for this protocol are RIB, EKSR & GT. RRJG, AMM and MCRF wrote the manuscript. RIB & MCRF created the protocol. VMCE & RRJG directed the publication. NM & MCRF did the methodological quality assessment. All the authors read and approved the final manuscript.

## Pre-publication history

The pre-publication history for this paper can be accessed here:

http://www.biomedcentral.com/1471-2474/13/248/prepub
